# First Robotic Roux-en-Y Gastric Bypass for the Treatment of Refractory Gastroesophageal Reflux Disease in a Patient With Systemic Sclerosis

**DOI:** 10.7759/cureus.33861

**Published:** 2023-01-17

**Authors:** Rodrigo Cañada T Surjan, Sergio Silveira, Estela R Figueira

**Affiliations:** 1 Surgery, Faculdade de Medicina da Universidade de São Paulo, São Paulo, BRA; 2 Surgery, Dasa/Hospital Nove de Julho, São Paulo, BRA; 3 Oncological Surgery, Dasa/Hospital Nove de Julho, São Paulo, BRA; 4 Gastroenterology, University of São Paulo Medical School, São Paulo, BRA

**Keywords:** roux-en-y, roux-en-y gastric bypass, gerd, systemic sclerosis, gastroesophageal reflux disease, esophageal motility disorders, robotic surgical procedures, gastric bypass, gastroesophageal reflux, systemic scleroderma

## Abstract

Systemic sclerosis (SSc) is an immune-mediated disease that results in fibrosis of the skin and internal organs. Refractory gastroesophageal reflux disease (GERD) associated with severe esophageal dysmotility is common in SSc patients, and surgical treatment with usual anti-reflux procedures such as fundoplications is associated with dismal symptomatic relief and postoperative dysphagia. We report the first robotic short-limb Roux-en-Y gastric bypass (RYGB) with a short Roux limb for the treatment of GERD in a patient with SSc with intense esophageal dysmotility. The operative time was two hours. The procedure and postoperative course were uneventful. The patient presented complete relief of gastroesophageal reflux symptoms and no postoperative dysphagia in a two-year follow-up. Therefore, short-limb RYGB is a safe and very effective alternative for the treatment of severe GERD in patients with SSc. The robotic surgical platform may have some advantages compared to conventional laparoscopy.

## Introduction

Systemic sclerosis (SSc) is a rare, immune-mediated, rheumatic, connective tissue disease that occurs more frequently in females aged 40-65 years characterized by abnormal collagen deposition and vasculopathy with subsequent fibrosis of the skin and internal organs [1,2]. It presents higher mortality than most rheumatic diseases that are associated with internal organ disease and usually diagnosis is delayed [3].

The most common initial clinical manifestations are Raynaud's phenomenon and gastroesophageal reflux disease (GERD), and the esophagus is the most frequent digestive system organ affected in up to 90% of the patients [4]. Other common manifestations of esophageal involvement by SSc are dysphagia and chest pain secondary to esophageal dysmotility [2].

Patients with SSc tend to have more severe GERD symptoms and reflux esophagitis [5]. They are more prone to develop complications such as Barrett's esophagus, peptic strictures, and more severe and faster aggravation of SSc underlying interstitial lung disease by recurrent aspiration with subsequent respiratory tree inflammation, and the gastrointestinal involvement-associated mortality of SSc patients is reported to be as high as 12% [6].

Surgical treatment of severe GERD refractory to drug therapies in SSc patients is a harsh challenge. Usual anti-reflux procedures, such as Nissen fundoplication, are associated with mediocre results, due to intense esophageal dysmotility in SSc patients [2]. Up to 71% of the SSc patients submitted to Nissen fundoplication will develop severe postoperative dysphagia and mild improvement in reflux symptoms [2].

On the other hand, Roux-en-Y gastric bypass (RYGB) has been described as an effective alternative for the treatment of acid reflux [7]. Recently, one study described the laparoscopic RYGB similar to the bariatric operation but performed with a shorter Roux limb as an efficient alternative to usual fundoplication (Nissen, Toupet, and Dor) in patients with SSc, with better relief of reflux symptoms and less postoperative dysphagia [8].

We describe the first robotic short-limb RYGB for the treatment of severe and refractory GERD that resulted in excellent symptomatic relief and no dysphagia in two years follow-up.

## Technical report

A 57-year-old female patient with a previous history of SSc diagnosed two years before and not in the use of immunosuppressant agents and cholecystectomy presented with recalcitrant GERD and failure of antisecretory medications to control reflux symptoms. Her body mass index was 23.

**Figure 1:**
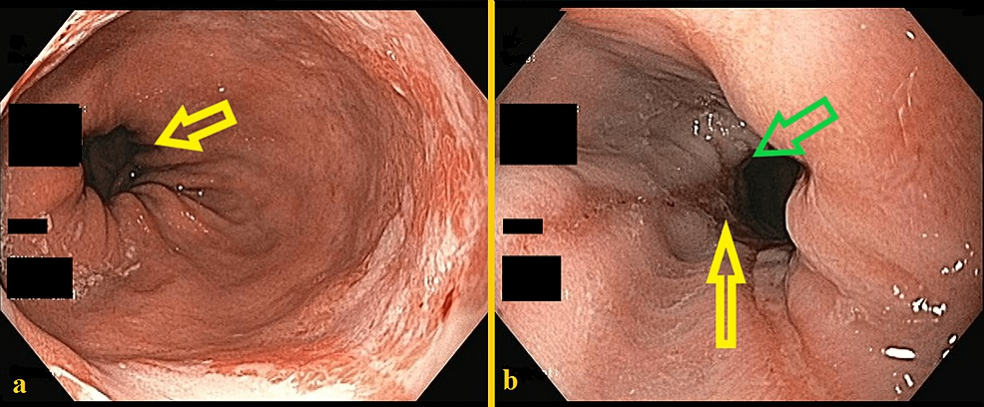
Upper digestive endoscopy (A) Hiatal hernia (yellow arrow: diaphragmatic compression). (B) Grade C esophagitis (yellow arrow). Green arrow: transition line between gastric and esophagic epithelium.

She was submitted to an upper digestive endoscopy that disclosed grade C esophagitis (Los Angeles classification) and a 3 cm sliding hiatal hernia (Figure 1).

An esophageal manometry disclosed lower esophageal sphincter hypotonia and absence of contraction in the distal two-thirds of the esophagus (Table 1).

**Table 1:** High-resolution esophageal manometry Esophageal manometry report values disclosing the failure of all 12 swallows with an absence of contraction of the distal esophagus. IRP: integrated relaxation pressure; DCI: distal contractile integral.

# Swallows	IRP (mmHg)	DCI (mmHg.s.cm)	Vigor
1	2.1	0.0	Failure
2	1.0	0.0	Failure
3	0.7	0.0	Failure
4	0.9	0.0	Failure
5	0.0	0.0	Failure
6	0.0	2.2	Failure
7	1.2	0.0	Failure
8	1.2	0.0	Failure
9	0.7	0.0	Failure
10	0.8	0.0	Failure
11	1.3	0.0	Failure
12	1.5	0.0	Failure

**Figure 2:**
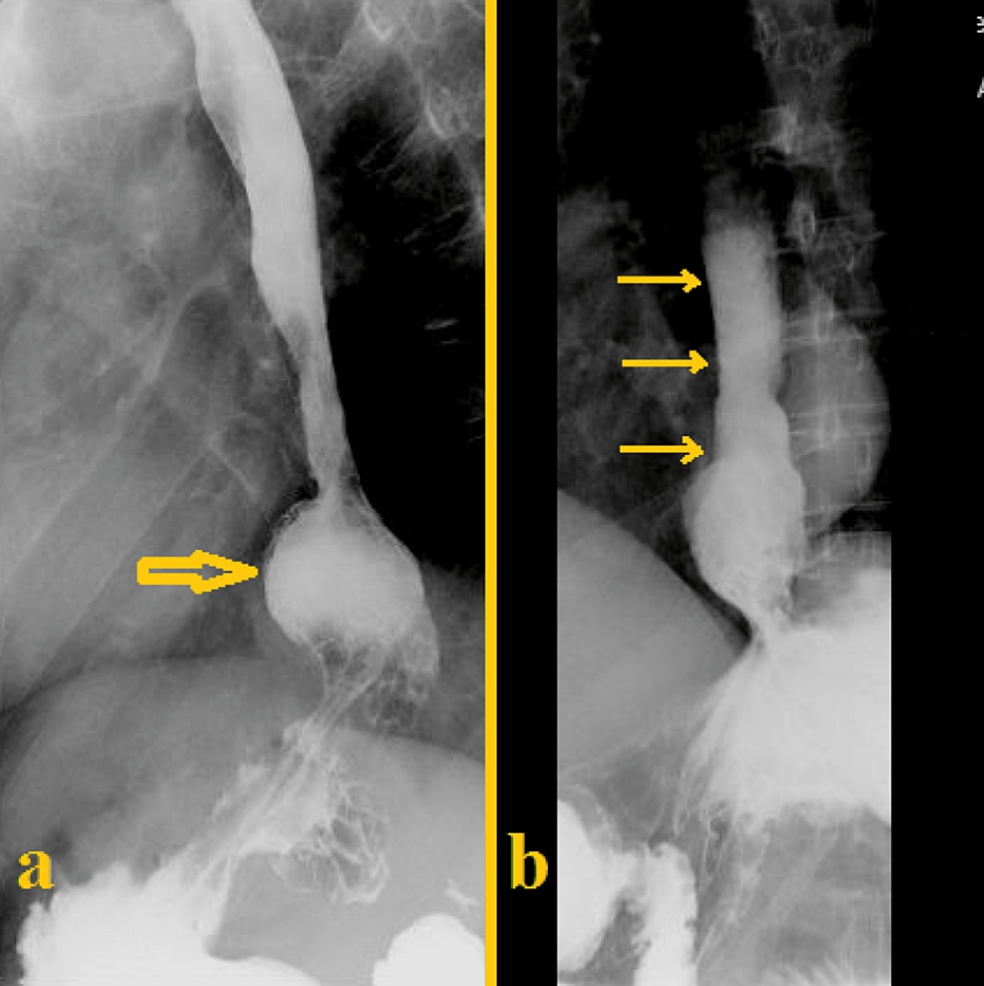
Barium esophagogram (A) Hiatal hernia (yellow arrow). (B) Gastroesophageal reflux (yellow arrows).

A barium esophagogram was performed and disclosed signs of esophageal dyskinesia, moderate hiatal hernia, and gastroesophageal reflux (Figure 2).

As fundoplication techniques are associated with poor outcomes regarding reflux symptoms relief and severe postoperative dysphagia in patients with SSc, a totally robotic RYGB with a short Roux limb associated with a hiatal closure was performed.

**Figure 3:**
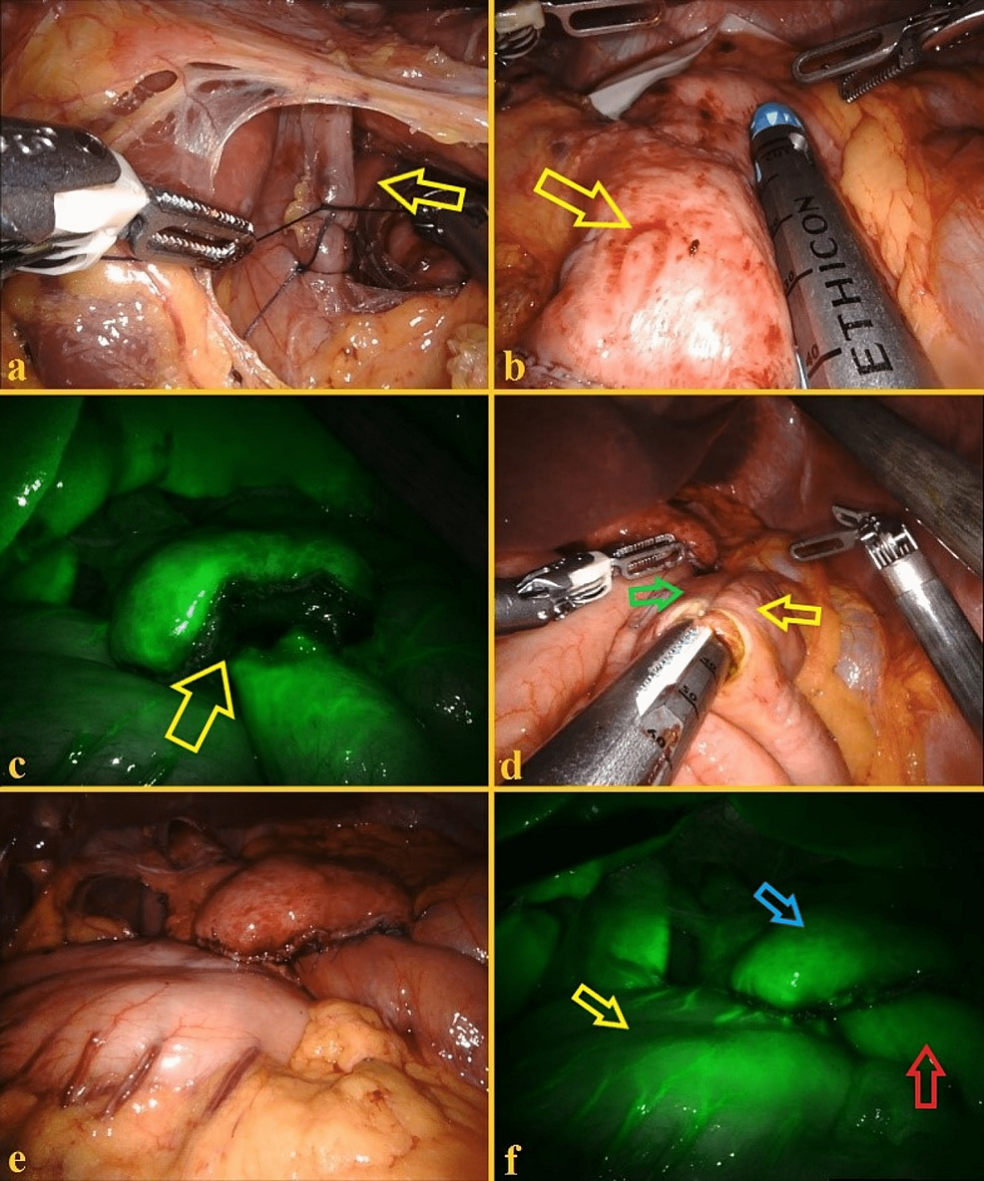
Intraoperative images (A) Closure of the esophageal hiatus (yellow arrow). (B) Creating the gastric pouch (yellow arrow). (C) Real-time near-infrared robotic fluorescence evaluation of the gastrojejunostomy (yellow arrow) with vascular perfusion using intravenous indocyanine green as a contrast agent. (D) Mechanical jejunojejunostomy between the biliopancreatic limb (green arrow) and the Roux limb (yellow arrow) at 35 cm of the Roux limb from the gastrojejunostomy. (E) Final aspect. (F) Final fluorescence evaluation of the vascular perfusion of the bypassed stomach (yellow arrow), gastric pouch (blue arrow), Roux limb (red arrow), and gastrojejunostomy.

The procedure began like a Nissen fundoplication, with retraction of the left liver sector, opening of the gastrohepatic ligament (preserving an aberrant left hepatic artery and the Latarjet nerve), dissection of the phrenoesophageal membrane, and exposure of the right crus. The dissection was carried out until the left crus was identified. A careful dissection of the left crus and angle of His was performed. Then, after exposing the crura for about 3 cm, the hiatus was closed using two 3-0 separate Vicryl sutures (Figure 3A).

**Figure 4:**
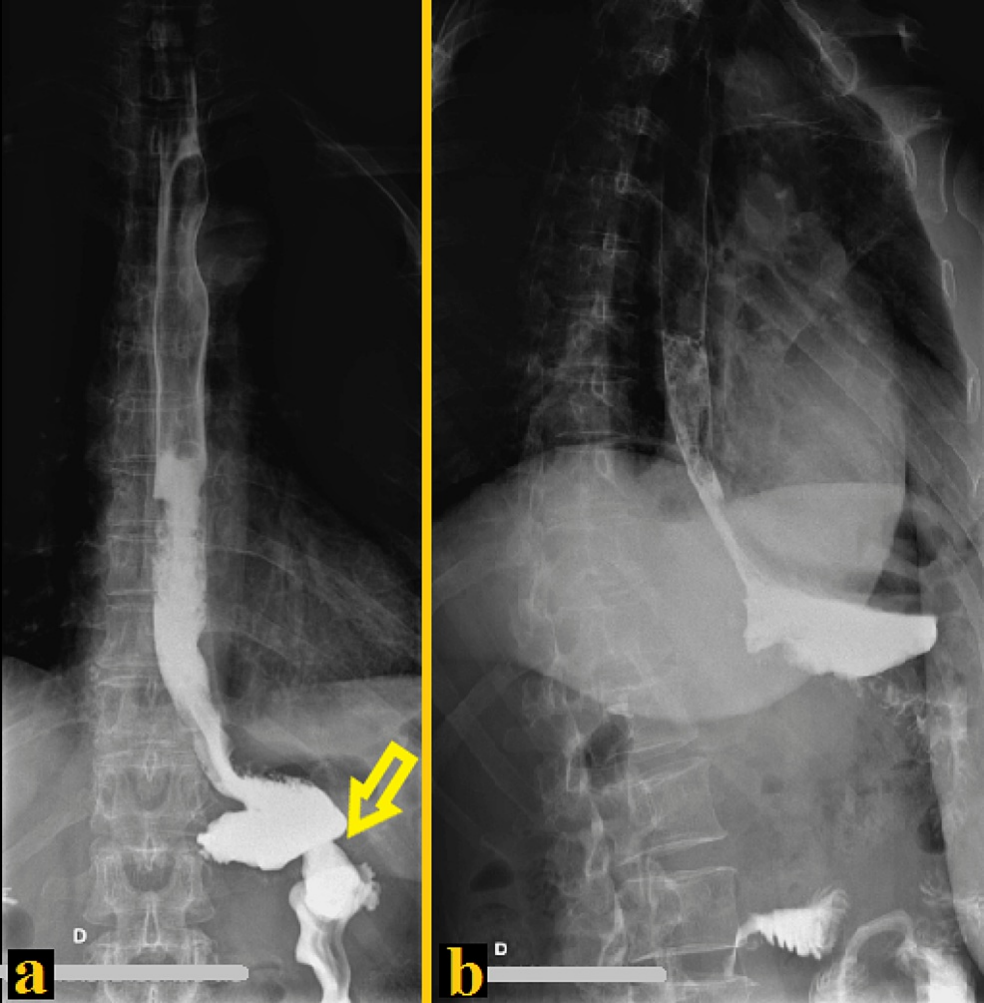
Postoperative day two upper gastrointestinal series with Gastrografin disclosing normal esophageal emptying, open gastroenteric anastomosis (yellow arrow), and no evidence of gastroesophageal reflux (A) Coronal plane. (B) Oblique plane.

At this time, the stomach was divided using an endoscopic vascular stapler to create a gastric pouch bigger than the usual 15 milliliters pouches performed during bariatric gastric bypass (Figure 3B). After that, about 40 cm of the jejunum was measured from the ligament of Treitz and divided with an endoscopic stapler. Then, a mechanical gastrojejunostomy was performed between the posterior aspect of the gastric pouch and the Roux limb. Among several advantages of the robotic approach over the laparoscopic approach, we performed real-time near-infrared with intravascular indocyanine green enhanced fluorescence to evaluate vascular perfusion of the gastric pouch, the Roux-en-Y jejunal loop, and the final anastomosis (Figure 3C). Finally, the jejunojejunostomy between the biliopancreatic limb and the Roux limb was performed mechanically with an endoscopic stapler, after measuring only 35 cm from the gastrojejunostomy in the Roux limb, to create a short-limb gastric bypass (Figure 3D). At the end of the procedure (Figure 3E), once again a real-time fluorescence using intravenous indocyanine green was performed to certify vascular perfusion of the bypassed stomach, Roux limb, gastric pouch, and gastrojejunostomy (Figure 3F). No abdominal drains were placed. The operative time was two hours. The postoperative period was uneventful. A full liquid diet was initiated one day after the procedure, pureed diet on postoperative day three, and soft food was initiated two weeks after the surgery. On postoperative day two, the patient was submitted to an upper gastrointestinal series with Gastrografin that disclosed normal esophageal anatomy, normal esophageal emptying, open gastroenteric anastomosis, and no evidence of gastroesophageal reflux (Figure 4).

The patient was discharged from the hospital on the fourth postoperative day. Two years after the procedure, the patient presents complete relief of GERD-related symptoms and no dysphagia.

## Discussion

GERD is a common clinical manifestation of SSc and tends to be more severe and results in life-threatening complications in these patients. SSc patients often develop GERD symptoms refractory to medical treatment, leaving the option for surgical treatment [6].

Most of the time, and as a result of the systemic disease, SSc patients have intense esophageal dysmotility, and conventional surgical techniques such as fundoplications result in intense postoperative dysphagia and dismal symptomatic relief [2].

In the scenario, RYGB with a short Roux limb emerges as an alternative treatment for severe GERD in SSc patients [7]. We report the first procedure performed by a totally robotic approach, and the patient presented complete symptomatic relief and no dysphagia two years after the surgery.

The robotic surgical platform has several well-known advantages over the laparoscopic approach, such as better ergonomics, high-definition tridimensional imaging, seven motion degrees, stable camera positioning, and tremor filtering [9]. One possible advantage of the robotic platform for RYGB is a lower incidence of anastomotic leak compared to conventional laparoscopy. Specifically related to our particular procedure, the robotic platform also allowed the evaluation of the vascular perfusion of the gastric pouch and gastroenteral anastomosis with real-time near-infrared fluorescence after intravenous injection of indocyanine green as a contrast agent. This may be a useful tool in evaluating the risk and preventing the development of postoperative anastomotic leaks [10].

We consider that the innovations proposed by our technique were the creation of a short Roux limb to avoid significant postoperative weight loss, the use of the robotic platform to perform this technique to specifically treat GERD in an SSc patient, and the use of indocyanine green fluorescence to evaluate the perfusion of the gastrojejunal anastomosis.

The limitation of this study is that this is a report of a single patient. Therefore, strong conclusions based on statistical analysis are not possible. While RYGB is not an innovative indication of surgical treatment for refractory GERD in SSc, we consider that the use of a short Roux limb, the use of the robotic platform confirming its well-known and reported technical advantages, and fluorescence perfusion evaluation were possible factors involved in the excellent postoperative outcome.

## Conclusions

A modified RYGB with a short Roux limb may be a more effective alternative to usual fundoplications for the treatment of recalcitrant GERD in SSc patients with esophageal motility disorder. We report the first totally robotic RYGB performed in an SSc patient with severe GERD.
